# Poor Neutralizing Antibody Responses in 132 Patients with CLL, NHL and HL after Vaccination against SARS-CoV-2: A Prospective Study

**DOI:** 10.3390/cancers13174480

**Published:** 2021-09-06

**Authors:** Evangelos Terpos, Maria Gavriatopoulou, Despina Fotiou, Chara Giatra, Ioannis Asimakopoulos, Maria Dimou, Aimilia D. Sklirou, Ioannis Ntanasis-Stathopoulos, Ismini Darmani, Alexandros Briasoulis, Efstathios Kastritis, Maria Angelopoulou, Ioannis Baltadakis, Panayiotis Panayiotidis, Ioannis P. Trougakos, Theodoros P. Vassilakopoulos, Maria Pagoni, Meletios A. Dimopoulos

**Affiliations:** 1 Department of Clinical Therapeutics, School of Medicine, National and Kapodistrian University of Athens, 11528 Athens, Greece; mgavria@med.uoa.gr (M.G.); desfotiou@med.uoa.gr (D.F.); johnntanasis@med.uoa.gr (I.N.-S.); abriasoulis@med.uoa.gr (A.B.); ekastritis@med.uoa.gr (E.K.); mdimop@med.uoa.gr (M.A.D.); 2 BMT Unit, Department of Hematology and Lymphomas, Evangelismos General Hospital, 10676 Athens, Greece; xgiatra@med.uoa.gr (C.G.); isminidarmani@gmail.com (I.D.); ibaltadakis@icloud.com (I.B.); marianpagoni@yahoo.com (M.P.); 3 Department of Hematology and Bone Marrow Transplantation, National and Kapodistrian University of Athens, Laikon General Hospital, 11527 Athens, Greece; iasimak@med.uoa.gr (I.A.); mkangelop@med.uoa.gr (M.A.); tvassilak@med.uoa.gr (T.P.V.); 4 First Propedeutic Department of Internal Medicine, National and Kapodistrian University of Athens, Laikon General Hospital, 11527 Athens, Greece; msdimou@med.uoa.gr (M.D.); ppanayi@med.uoa.gr (P.P.); 5 Department of Cell Biology and Biophysics, Faculty of Biology, National and Kapodistrian University of Athens, 15784 Athens, Greece; asklirou@biol.uoa.gr (A.D.S.); itrougakos@biol.uoa.gr (I.P.T.)

**Keywords:** SARS-CoV-2, COVID-19 vaccination, BNT162b2 vaccine, chronic lymphocytic leukemia, non-Hodgkin’s lymphoma, Hodgkin’s lymphoma, humoral immune response, Rituximab, Bruton’s tyrosine kinase inhibitors, neutralizing antibodies

## Abstract

**Simple Summary:**

The urgency of the COVID-19 pandemic has led to accelerated vaccine development within less than a year. Emerging data suggest that the ability of patients with hematological malignancies to form an adequate number of antibodies in response to vaccination for SARS-CoV-2 is suboptimal. In this context, we evaluated the ability of 132 patients with Chronic Lymphocytic Leukemia, Non-Hodgkin’s Lymphoma and Hodgkin’s Lymphoma to elicit an adequate immune response to the BNT162b2 vaccine. Vaccination with two doses of the BNT162b2 vaccine led to lower production of neutralizing antibodies against SARS-CoV-2 in these patients compared with healthy controls. Being on active treatment for the underlying disease was an independent prognostic factor for suboptimal antibody response. This finding underlines the need for timely vaccination ideally during a treatment-free period and for continuous vigilance on infection control measures.

**Abstract:**

Emerging data suggest suboptimal antibody responses to COVID-19 vaccination in patients with hematological malignancies. We evaluated the humoral response following the BNT162b2 vaccine in patients with chronic lymphocytic leukemia (CLL), non-Hodgkin’s lymphoma (NHL), and Hodgkin’s lymphoma (HL). An FDA-approved, ELISA-based methodology was implemented to evaluate the titers of neutralizing antibodies (NAbs) against SARS-CoV-2 on day 1 of the first vaccine, and afterwards on day 22 and 50. One hundred and thirty-two patients with CLL/lymphomas and 214 healthy matched controls vaccinated during the same period, at the same center were enrolled in the study (NCT04743388). Vaccination with two doses of the BNT162b2 vaccine led to lower production of NAbs against SARS-CoV-2 in patients with CLL/lymphomas compared with controls both on day 22 and on day 50 (*p* < 0.001 for all comparisons). Disease-related immune dysregulation and therapy-related immunosuppression are involved in the low humoral response. Importantly, active treatment with Rituximab, Bruton’s tyrosine kinase inhibitors, or chemotherapy was an independent prognostic factor for suboptimal antibody response. Patients with HL showed superior humoral responses to the NHL/CLL subgroups. In conclusion, patients with CLL/lymphomas have low humoral response following COVID-19 vaccination, underlining the need for timely vaccination ideally during a treatment-free period and for continuous vigilance on infection control measures.

## 1. Introduction

The worldwide pandemic declared in March 2020, caused by the spread of the novel coronavirus, severe acute respiratory syndrome coronavirus 2 (SARS-CoV-2), remains an ongoing global health issue [1,2]. COVID-19 is a multisystemic disease with short- and long-term manifestations [3,4]. The clinical spectrum ranges from mild symptoms to a severe and life-threatening disease course in up to 5–10% of patients [3]. The urgent need to achieve herd immunity has made vaccine development a global priority [5,6] and led to accelerated vaccine development in less than a year [5,7]. Significant efficacy has been demonstrated for different vaccine types in several phase 3 placebo-controlled, randomized trials (RCTs). The safety, efficacy, and durability of the different vaccines in a real-world setting are currently under investigation [8]. In addition to the above, patients with hematological malignancies are underrepresented in clinical trials [7,9].

The risk of severe disease presentation, complications, and worse outcomes is higher amongst immunocompromised patients with hematological malignancies compared to the general population and the risk of death amongst hospitalized patients is as high as 39% [10,11,12]. Moreover, higher morbidity and mortality are reported compared with patients with solid organ tumors [12]. Importantly, all patients with solid and hematological cancers achieve lower seroconversion rates following COVID-19 infection compared to non-cancer patients [13,14,15,16,17].

Patients with lymphoproliferative disorders including chronic lymphocytic leukemia (CLL), non-Hodgkin (NHL), and Hodgkin’s lymphoma (HL) (CLL/NHL/HL or CLL and all kinds of lymphomas—CLL/Ly) are at increased risk for severe COVID-19 disease and death due to the immunocompromised status associated with the underlying disease, older age, and comorbidities [18,19,20,21,22,23]. In addition to measures such as mask-wearing, social distancing, and modifications in treatment schedules, vaccination constitutes one of the most important preventive strategies amongst patients with CLL/Ly [24,25]. The efficacy of COVID-19 vaccines in this population remains, however, largely unknown [7]. Emerging data suggest that patients with lymphoproliferative disorders including CLL/Ly patients [16,26,27] are likely to present decreased antibody responses following COVID-19 vaccination. The suboptimal immune response to vaccination is most likely multifactorial and linked to defects in immune effectors cells [28] associated with the underlying B-cell pathology and the effects of treatment agents such as anti-CD20 antibodies and Bruton’s tyrosine kinase inhibitors [28]. 

Herein, we describe the humoral response, as indicated by the development of neutralizing antibodies (NAbs), against SARS-CoV-2 in patients with CLL/HL/NHL after vaccination with the mRNA BNT162b2 vaccine up to day 50 (D50) post their first vaccine dose. In addition, we sought to evaluate possible interactions with clinical characteristics and treatment data.

## 2. Results

### 2.1. Baseline Characteristics of Patients and Controls

The study population included 132 patients (6 males (50%)/66 females (50%); mean age: 64.6 yrs, SD: ±14.3) and 214 controls (96 males (44.9%)/118 females (55.1%); mean age: 69.8 yrs, SD: ±12.5). All participants were vaccinated during the same period (1st January 2021–31st May 2021), at the same vaccination center (Alexandra General Hospital, Athens, Greece). The patients were vaccinated based on the Greek vaccination program. All patients and controls were vaccinated with the BNT162b2 vaccine. 

The characteristics of the patients with CLL/Ly are depicted in Table 1. At the time of vaccination, 45 (48.9%) out of 92 symptomatic patients were receiving therapy, 47 (51.1%) were in remission after prior treatment and did not receive any therapy at the time of vaccination, whereas 40 out of 132 (30%) patients had asymptomatic disease without current or prior treatment. Active treatment was defined as CLL and lymphoma-specific treatment with either chemotherapy or immunotherapy or targeted therapy and their combinations in the last 30 days. Among the patients with symptomatic CLL/Ly without active treatment, the median time from the last treatment dose was 14.5 (range 2–154) months.

### 2.2. Humoral Response in CLL/Ly Patients and Controls

**Figure 1:**
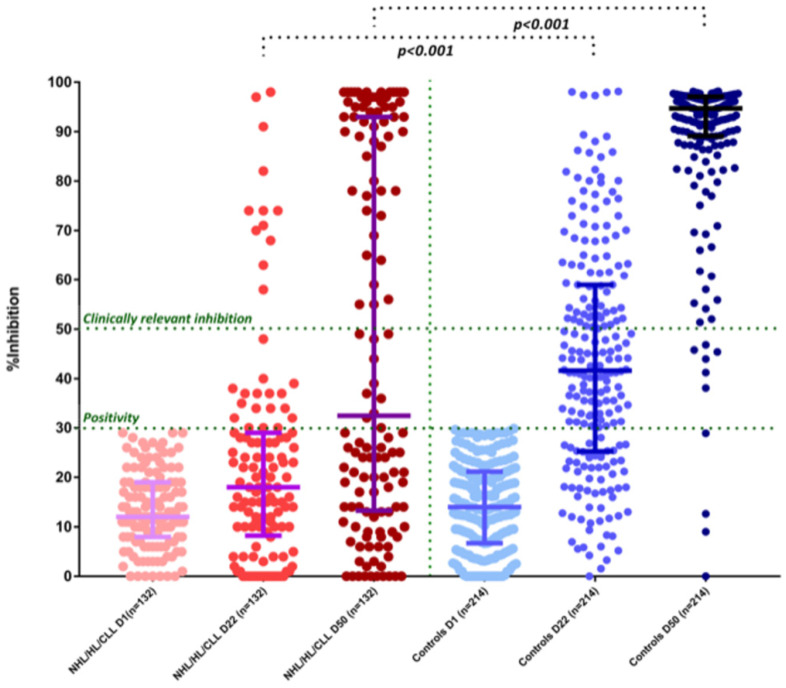
Kinetics of NAbs in CLL/lymphoma patients compared with controls after vaccination with 2 doses of the BNT162b2 (post hoc mixed-model repeated-measures analysis). On D22, patients had lower NAb inhibition titers compared with controls (see text). Only 12/132 (9%) patients had NAb titers of equal or more than 50%. Similarly, patients had lower NAb inhibition titers compared with controls on D50 (see text). Only 58/132 (43.9%) patients had NAb titers of equal or more than 50%.

On day 1 (D1), no patients or controls had NAb titers of ≥30% (positivity cut-off) and none of them reported a prior history of known COVID-19. After the first dose of the vaccine, on day 22 (D22), CLL/Ly patients had lower NAb titers compared with controls: the median NAb inhibition titer was 18% (IQR: 8.5–29%) for CLL/Ly patients versus 41.6% (IQR: 25.3–59%) for controls; *p* < 0.001 (Figure 1). More specifically, only 22% (29/132) of the patients versus 71% (152/214) controls developed NAb titers ≥30% on D22 (*p* < 0.001). The respective number of patients and controls who developed NAb titers ≥50% was 9% (12/132) and 28% (81/214), respectively (*p* < 0.001) (Figure 1).

After the second dose of the vaccine, on D50, CLL/Ly patients had lower NAb titers compared with controls. The median NAb inhibition titer was 32.5% (IQR: 13.5–93%) for patients versus 94.7% (IQR: 89–97%) for controls; *p* < 0.001 (Figure 1). More specifically, only 50.8% (67/132) of the patients versus 98.1% (210/214) of the controls developed NAb titers ≥30% on D50 (*p* < 0.001). The respective number of patients and controls who developed NAb titers ≥50% was 43.9% (58/132) and 95.3% (204/214) (*p* < 0.001) (Figure 1). Among these high-responders (*n* = 58) 37 (63.7%) patients had symptomatic disease and 7 (12%) were on active treatment; 2 patients were on ibrutinib monotherapy, 2 receiving a rituximab-combination, 1 patient receiving venetoclax monotherapy, and 2 a chemotherapy-only based regimen. Among responders on active treatment, 2 patients had CLL, 3 NHL, and 2 HL. Among the 30 patients who were in remission (complete or partial) and not on active therapy, the median time off-treatment was 19.5 months (3–129 months). Among patients who achieved clinically relevant humoral response (NAb titers ≥50%) only 7 patients had the uninvolved immunoglobulins out of normal limits (4 patients decreased IGA, and 3 decreased both IGG and IGA).

Among the patients with low response rates (<30%) at day 50 (*n* = 74), 54 were symptomatic and 19 asymptomatic. Among the 54 symptomatic patients with CLL/Ly, 37 were on active treatment at the time of vaccination; 4 patients were receiving BTKi with Rituximab, 16 under monotherapy with a Bruton’s Tyrosine Kinase (BTK) inhibitor, 14 patients under treatment with a Rituximab-based regimen, and 3 patients receiving a chemotherapy-based regimen only. The other patients were previously treated but not on active treatment.

### 2.3. Predictive Factors for NAb Production

There were no significant differences at baseline regarding NAb levels between patients and healthy controls. Patients with CLL/Ly showed an inferior NAb response in all subsequent time points (D22 and D50) compared with controls (*p* < 0.05 for all comparisons). 

**Figure 2:**
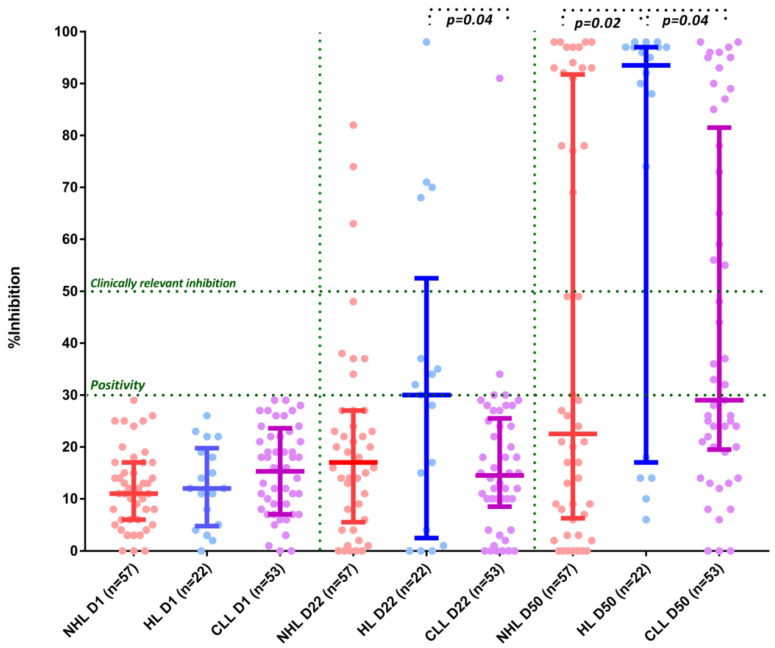
Kinetics of NAbs in non-Hodgkin’s lymphoma (NHL) patients vs Hodgkin’s lymphoma (HL) patients vs chronic lymphocytic leukemia (CLL) patients after vaccination with 2 doses of BNT162b2 (post hoc mixed-model repeated-measures analysis). On D22, patients with HL had significantly higher NAb inhibition titers compared with CLL patients (see text). On D50, patients with HL had higher NAb inhibition titers compared with both NHL and CLL patients (see text).

In the group of CLL/Ly patients, there was no statistically significant difference between the symptomatic and the asymptomatic patient subgroup at D50, respectively (40.7% versus 66.7%, *p* = 0.37). There was an interaction between the type of hematological malignancy (NHL vs. HL vs. CLL) and NAbs production at D22 (17% vs. 30% vs. 14.5%, *p* = 0.2 NHL vs. HL, *p* = 0.04 HL vs. CLL, *p* = 0.8 NHL vs. CLL respectively) and D50 (22.5% vs. 93.5% vs. 29%, *p* = 0.02 NHL vs. HL, *p* = 0.04 HL vs. CLL, *p* = 0.44 NHL vs. CLL) (Figure 2). Only 4 out of the 22 patients with HL were receiving treatment at the time of vaccination.

Among patients with symptomatic disease, only 4 (12.5%) patients on active treatment had >50% NAbs at D50 compared with 32 symptomatic patients who did not receive active treatment at the time of vaccination (57.1%) (*p* < 0.001). Regarding the patients on active treatment at day 22, there was no difference among the treatment groups regarding the humoral response (BTK-only versus Rituximab-only versus BTK with rituximab versus chemotherapy; median NAbs 15% versus 20% versus 13% versus 17.5%, respectively, *p* = NS), as well as D50 (29% versus 15.5% versus 5% versus 85%, respectively, *p* = NS, numerical advantage for chemotherapy arm). The subgroup of symptomatic patients who were treated with Rituximab-based combinations in the last 12 months and achieved humoral responses of at least 50% at day 50 were 6/28 (21.4%) versus 31/63 (49.2%) for patients who had never received rituximab or had received rituximab more than 12 months ago, respectively (*p* = 0.01). 

No correlations were identified between gender, body mass index (BMI), prior lines of therapy, lymphocyte number, gamma globulins, and levels of NAb production in CLL/Ly patients. (Table 2) On the contrary, active treatment and rituximab administration in the last 12 months correlated with decreased antibody response at day 50 (OR: 0.14, 95% CI: 0.06–0.35, *p* < 0.001 for active treatment and OR: 0.27, 95% CI: 0.1–0.71, *p* = 0.008 for rituximab). Compared with HL, NHL and CLL patients had decreased antibody response at day 50 (OR: 0.21, 95% CI: 0.06–0.69, *p* = 0.001 and OR: 0.22, 95% CI: 0.07–0.73, *p* = 0.01, for NHL and CLL, respectively).

Among CLL/Ly patients, the multivariable logistic regression (Table 2) adjusted for active treatment, administration of rituximab in the past 12 months, and disease type, showed that active treatment was significantly associated with lower antibody responses at day 50 (<50%) (OR: 0.15, 95% CI: 0.05–0.42, *p* < 0.001), whereas patients with HL were more likely to achieve higher humoral responses (>50% at day 50) compared with other disease types (OR: 4.9, 95% CI: 1.29–18.4, *p* = 0.019). There was a trend towards lower antibody response among those treated with rituximab in the last 12 months (OR: 0.33, 95% CI: 0.1–1.1, *p* = 0.07).

### 2.4. Adverse Events

**Figure 3:**
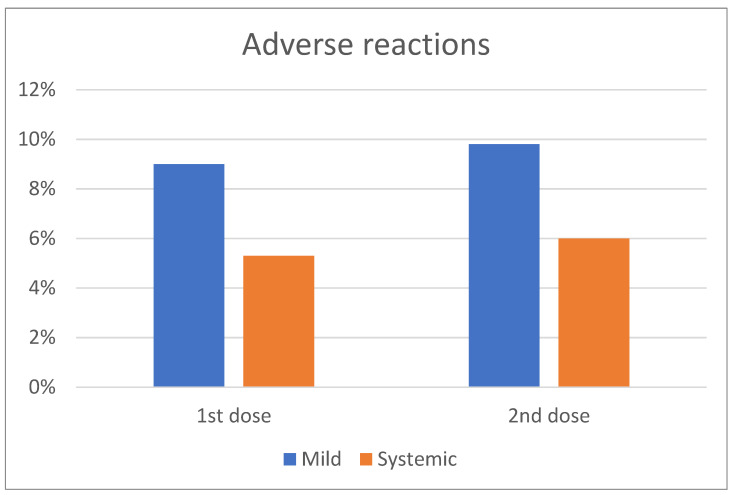
Frequency of mild and systemic adverse events following the first and second dose of COVID-19 vaccine.

Among patients with CLL/Ly, 11/132 (9%) and 13/132 (9.8%) reported mild reactions after the first and second dose of the BNT162b2 vaccine (Figure 3). These reactions included mainly pain at the site of the injection, erythema, and/or swelling. The rate of this adverse event between the first and second dose of the BNT162b2 was not statistically significantly different. 5.3% (7/132) and 6% (8/132) of the patients vaccinated with the BNT162b2 vaccine reported systemic adverse reactions after the first and second vaccine shot, respectively, which included fatigue, fever, and lymphadenopathy (Figure 3). The emergence of the adverse events related to vaccination was independent of the active treatment or disease status. Importantly, the adverse event rate was not different at any time point (first or second vaccine shot) between patients and controls.

## 3. Discussion

Our data indicate that vaccination with the BNT162b2 mRNA vaccine leads to lower production of NAbs against SARS-CoV-2 in patients with symptomatic and asymptomatic CLL, non-Hodgkin’s lymphoma and Hodgkin’s lymphoma compared with controls of similar age and gender without malignant disease. The strongest predictive factor for poor humoral responses was receiving active treatment for the underlying hematological malignancy at the time of vaccination. In addition, patients who had received Rituximab within the last 12 months had lower NAb titers compared to patients who had completed treatment for more than 12 months or had never received Rituximab. Finally, patients with HL achieved better humoral responses compared to the NHL and CLL subgroups and CLL/NHL remained an adverse prognostic factor for adequate humoral responses in the multivariate analysis.

Vaccination against SARS-CoV-2 is considered the most promising preventive strategy against COVID-19. Its efficacy, however, in patients with hematological malignancy has not yet been demonstrated and emerging data question the ability of these patients to elicit satisfactory humoral responses and establish adequate antibody titers [7]. 

The efficacy of the BNT162b2 mRNA vaccine against SARS-CoV-2 in healthy adults has been clearly demonstrated [29]. BNT162b2 is a nucleoside-modified RNA vaccine that is nanoparticle formulated and encodes a prefusion-stabilized membrane-anchored SARS-CoV-2 full-length spike protein. The effectiveness of the first BNT162b2 has been demonstrated in health care workers and octogenarians [30,31,32,33]. Among patients with lymphoproliferative disorders, antibody-mediated responses are much lower [16,26,27]; among 167 patients with CLL, only 39.5% had a positive antibody-mediated response to the BNT162b2 vaccine [27]. 

The suboptimal humoral response of CLL/Ly patients to vaccines can be attributed to underlying defects of the immune effector cells. The dysregulation of the immune system is multifactorial and secondary to the underlying B-cell disorder and pathology of the B-cell and the immunomodulatory effects of treatment administered [27,28,32,34]. Another point to consider may pertain to the co-existence of Epstein-Barr Virus (EBV) infection in patients with lymphomas and CLL, which may lead to suboptimal humoral responses. However, such data was not available in our study in order to investigate this aspect. 

B-cell depletion may impair the immune response to vaccines. Being on active treatment was the strongest independent adverse prognostic factor associated with poor response rates to the vaccine for all patients at both day 22 and day 50. Patients with HL were more likely to achieve humoral responses compared to NHL and CLL patients which remained an independent favorable prognostic factor in multivariate analysis. Hyperglobulinemia might be associated with inferior antibody responses among patients with CLL and COVID-19 [16]. The reconstitution of humoral immunity when patients are in response and not on active treatment is most likely a major determinant of effective antibody responses to the vaccine as demonstrated in our group of patients. 

Patients treated with BTK-inhibitors, venetoclax, and/or anti-CD20 antibodies are unlikely to respond to a single dose of vaccine. The humoral response to the vaccine is most likely impaired when patients receive treatment with BTK inhibitors which block B-cell receptor signaling in both malignant and normal B-cells [35,36,37,38]. Anti-SARS-CoV-2 NAb production is suboptimal in patients with CLL/Ly in analogy to the low response rates of 20–40% to the pneumococcal conjugate vaccine (PCV13), pneumococcal polysaccharide vaccine (PPSV23), HepB-CpG vaccine [38,39,40,41], as well as of the reduced efficacy of influenza A and B vaccination [41]. BTK inhibitors in particular are also associated with poor antibody-mediated response rates to influenza vaccination in patients with CLL [35,36] (rates of 7–26%) and decreased immune responses to the anti-hepatitis B vaccine, HepB-CpG19 [42]. In our cohort, humoral responses at day 22 and day 50 in the patient subgroup that received BTK inhibitors were lower compared to patients who received Rituximab-based or chemotherapy-only regimens, but the difference did not reach statistical significance. 

Exposure to anti-CD20 antibodies like Rituximab which lead to B-cell depletion also reduces the humoral response to influenza vaccine, the pneumococcal polysaccharide vaccine, and others [43]. In our study, patients with CLL/Ly treated with Rituximab in the last 12 months had suboptimal humoral responses compared to patients who had never received rituximab or had completed treatment more than 12 months prior to vaccination. Similar results have been described in patients with Waldenstrom’s macroglobulinemia under therapy with rituximab or BTK-inhibitors [44], as well as in patients with multiple myeloma under treatment with anti-CD38 or anti-BCMA-based regimens [45], suggesting that therapies targeting lymphocytes and plasma cells lead to poor antibody responses after vaccination against SARS-CoV-2.

Patients who had received multiple lines of treatment, and patients with CLL compared to lymphomas, had poor antibody responses after the first vaccination. The proportion of patients with adequate antibody titers increased considerably following the second booster dose. Our results, therefore, suggest that a second timely vaccine dose is necessary for patients with hematological malignancies that have immune homeostasis deregulation, and especially for the elderly [46]. A single study demonstrated however that a high-dose booster vaccination strategy may lead to improved rates of seroprotection to the influenza vaccine. The recent regulatory approval of a third vaccine shot at least one month post the second vaccination dose in patients with hematological cancer will further improve the anticipated protection. Furthermore, T-cell-mediated vaccine approaches against SARS-CoV-2 are under clinical investigation for patients with B-cell immune deficiencies (egNCT04954469).

Vaccination allows for a lower risk of COVID-19 severe disease; studies performed, however, were not designed to detect a mortality protection signal. Data for cancer patient subgroups are lacking [47]. NAbs have an important predictive value of immune protection from symptomatic COVID-19 and they are therefore considered valuable surrogates for the efficacy of the vaccine [48]. The effectiveness of SARS-CoV-2 vaccination against severe COVID-19 infection and the risk of reinfection by SARS-CoV-2 variants in patients with CLL/Ly remains unknown. 

## 4. Patients and Methods

### 4.1. Patients and Controls

Major inclusion criteria for the study included: (i) age above 18 years; (ii) presence of CLL/NHL/HL irrespective of the treatment phase, and (iii) eligibility for vaccination. Volunteer controls of similar age were also included in this analysis. We included healthy individuals without malignant disease above 60 years old vaccinated during the same time period (January–May 2021). The age cut-off was selected taking into consideration the median age of patients with CLL/NHL/HL [23] and the age groups that were prioritized according to the National Vaccination Program. Major exclusion criteria for both patients and controls included the presence of: (i) autoimmune disorders or active malignant disease besides CLL/NHL/HL; (ii) HIV or active hepatitis B and C infection, and (iii) end-stage renal disease. These disease entities were excluded due to concerns of confounding effect on antibody response following vaccination. Relevant data were extracted from the medical records and included: demographics, complete blood count, serum immunoglobulin (Ig) levels, disease status, and type of treatment. 

All participants (patients with CLL/Ly and controls) have been enrolled in a large prospective study (NCT04743388) evaluating the kinetics of anti-SARS-CoV-2 antibodies after COVID-19 vaccination in healthy subjects and patients with hematological malignancies or solid tumors. According to the National Vaccination Program in Greece, the two doses of BNT162b2 are administered three weeks apart. The study was approved by the Institutional Ethics Committee of General Hospital Alexandra, Athens, Greece, in accordance with the Declaration of Helsinki and the International Conference on Harmonization for Good Clinical Practice. All patients and controls provided written informed consent prior to enrollment in the study. 

### 4.2. NAbs Measurement

After vein puncture, the serum of both patients and controls was collected on day 1 (D1; before the first BNT162b2 dose), on day 22 (D22; before the second dose of the BNT162b2), and on day 50 (D50; 3 weeks post the second dose of the BNT162b2). Serum was separated within 4 h from blood collection and stored at −80 °C until the day of measurement. NAbs against SARS-CoV-2 were measured using an FDA-approved methodology (ELISA, cPass™ SARS-CoV-2 NAbs Detection Kit; GenScript, Piscataway, NJ, USA) [49] on the abovementioned timepoints, as previously described [33,50]. An NAb titer of at least 30% is considered positive, whereas an NAb titer of at least 50% has been associated with clinically relevant viral inhibition [45,51,52,53]. Samples of the same individual were measured in the same ELISA plate. 

### 4.3. Statistical Analysis 

All statistical analyses were performed with STATA (version 17.0, College Station, Texas). All variables were tested for normal data distribution. The analyses were performed on an intention-to-treat as well as on-treatment basis. Normally distributed data were expressed as the means ± standard deviation (SD). Non-normally distributed data were presented as the median with the interquartile range. For categorical variables, the Chi-square or Fisher exact tests were used to compare the distributions for the two randomized groups. Wilcoxon ranked samples were used for between-treatment comparisons of continuous variables. A post hoc mixed-model repeated-measures analysis was used to evaluate the neutralizing antibodies over time with cases and controls as main effects and neutralizing antibodies as dependent variables. Mixed models were performed using direct likelihood estimation with fixed effects of groups, time of antibodies, and interaction of groups (cases, controls) by the timing of antibody measurement. An unstructured covariance matrix was used to model within-patient error. We also used a multivariable linear regression model adjusted for active treatment, administration of rituximab for the past 12 months, and disease type in order to evaluate the effect of these factors on NAb production at D22 and D50. All variables were categorical. All significance tests were two-tailed and conducted at the 5% significance level.

## 5. Conclusions

The antibody-mediated responses to SARS-CoV-2 vaccination in patients with CLL/Ly are suboptimal, and humoral immunity seems to be deregulated. However, vaccination remains essential. Mucosal surface antibodies, such as IgA and T-cell responses, might be of equal importance in the immune responses after SARS-CoV-2 infection or vaccination [54,55]. Memory B-cell and T-cell responses may however also be compromised in patients with hematological malignancies [56]. Data specifically for this subpopulation is still missing and studies on the kinetics of the immune system following COVID-19 vaccination are required to shed light on the responses of the immune landscape in patients with B-cell malignancies. An important consideration is the effort to perform vaccination prior to treatment initiation when possible. Studies are required to evaluate different dosing, dosing intervals, and the number of boosting doses. In addition to the above, prioritization for booster doses in patients with suboptimal responses is essential [57]. Finally, long-term follow-up and close monitoring will allow the evaluation of potential concurrent or synergistic adverse events of the COVID-19 vaccine in these patients. 

## Figures and Tables

**Table 1:** Characteristics of patients with CLL/lymphoma.

Number of Patients (Male/Female, *n*)	*n* = 132 (66/66)
Age (yrs), mean ± SD	64.6 ± 14.3
NHL HL CLL (symptomatic/asymptomatic)	*n* = 57 (43%) *n* = 22 (17%) *n* = 53 (40%) (30/23)
Patients on active treatment at the time of vaccination	*n* = 45/132 (34%)
Type of therapy	Rituximab-BTKi: 4/45 (9%) Rituximab-based: 16/45 (35.5%) BTKi-based (ibrutinib, acalabrutinib): 18/45 (40%) Other (ABVD, bcl-2 inhibitor): 7/45 (15.5%)
Comorbidities	Pulmonary disease: 3.2% Diabetes mellitus: 14.4% Autoimmune disease: 1.6% Cardiovascular disease: *n* = 37%
Immunoglobulins, median, IQR mg/dl	IgG: 931 (799–1100) IgA: 106 (70–180) IgM: 35 (25–65)
Total lymphocyte count, median, IQR cells/mm^3^	1910 (1200–3400)

NHL: non-Hodgkin lymphoma, HL: Hodgkin lymphoma, CLL: chronic lymphocytic leukemia BTKi: Bruton tyrosine kinase inhibitor; ABVD: adriamycin, bleomycin, vincristine, dacarbazine; SD: standard deviation; IQR: interquartile range.

**Table 2:** Predictive factors for NAb production in patients as assessed in multivariate and univariate analysis.

Variables	Univariate (OR, 95% CI)	Multivariate * (OR, 95% CI)	*p*-Value
Age	0.99, 0.97–1	-	
Gender Male Female	(Reference) 1.34, 0.8–2.2	-	
Body Mass Index	1.03, 0.95–1.1	-	
Lymphopenia	0.96, 0.35–2.6	-	
Immunoparesis (decreased g-globulins)	0.56, 0.04–7.44	-	
Symptomatic disease	0.34, 0.3–3.9	-	
Disease type			
Non-Hodgkin lymphoma	(Reference)	(Reference)	
Hodgkin lymphoma	4.7, 1.4–15.6	4.9, 1.3–18.4	*p* = 0.019
Chronic lymphocytic leukemia	1.06, 0.46–2.4	0.96, 0.4–2.5	
Active treatment	0.14, 0.06–0.35	0.15, 0.05–0.42	*p* < 0.001
Rituximab in the last 12 months	0.27, 0.1–0.7	0.33, 0.1–1.1	*p* = 0.07
Treatment type No treatment Bruton’s tyrosine kinase inhibitors (BTKi) Rituximab Combination (BTKi-Rituximab) Chemotherapy	(Reference) 0.54, 0.2–1.5 0.2, 0.06–0.8 1, 0.8–1.2 1.24, 0.26–5.9	-	

Categorical variables: gender, symptomatic disease, disease type, active treatment, rituximab in the last 12 months, treatment type; * only statistically significant variables in the univariate analysis were included in the multivariate model.

## Data Availability

For original data, please contact eterpos@med.uoa.gr.
